# Supplementary material for: Hybrid Models and Biological Model Reduction with PyDSTool
Source: PLoS Comput Biol. 2012 Aug 9;8(8):e1002628. doi: 10.1371/journal.pcbi.1002628 (PMC3415397; doi:10.1371/journal.pcbi.1002628)
Supplement: Text S4 — Complete source code for the PyDSTool package (version 0.88.120504). Includes API documentation and help files linking to web pages. This file is identical to the current public release on Sourceforge.net. (ZIP) [file pcbi.1002628.s004.zip › PyDSTool/html/PyDSTool.Trajectory'-pysrc.html]

xml version="1.0" encoding="ascii"?


PyDSTool.Trajectory'


| Home | Trees | Indices | Help | PyDSTool |
| --- | --- | --- | --- | --- |

Package PyDSTool ::
Module Trajectory'

[hide private]

[frames] | no frames]

# Source Code for Module PyDSTool.Trajectory'

```
   1  """Trajectory classes.
 
   2  
 
   3     Robert Clewley, June 2005.
 
   4  """ 
   5  
 
   6  # ----------------------------------------------------------------------------
 
   7  
 
   8  # PyDSTool imports
 
   9  from Variable import * 
  10  from Interval import * 
  11  from Points import * 
  12  from utils import * 
  13  from common import * 
  14  from common import _num_types 
  15  from parseUtils import * 
  16  from errors import * 
  17  
 
  18  # Other imports
 
  19  from numpy import array, arange, float64, int32, concatenate, zeros, shape, \
 
  20       sometrue, alltrue, any, all, ndarray, asarray, Inf, unique 
  21  from scipy.optimize import bisect, newton 
  22  from numpy.linalg import norm 
  23  import math 
  24  import copy 
  25  import sys 
  26  
 
  27  # -------------------------------------------------------------
 
  28  
 
  29  ## Exports
 
  30  
 
  31  __all__ = ['Trajectory', 'HybridTrajectory', 'numeric_to_traj',
 
  32             'pointset_to_traj', 'convert_ptlabel_events', 'findApproxPeriod'] 
  33  
 
  34  # -------------------------------------------------------------
 
  35  
 
  36  
 


37 -def numeric_to_traj(vals, trajname, coordnames, indepvar=None, indepvarname='t',
 
  38                      indepdomain=None, all_types_float=True, discrete=True):


39      """Utility function to convert one or more numeric type to a Trajectory.
 
  40      Option to make the trajectory parameterized or not, by pasing an indepvar
 
  41      value.
 
  42  
 
  43      To use integer types for trajectory coordinates unset the default
 
  44      all_types_float=True argument.
 
  45  
 
  46      To create interpolated (continuously defined) trajectories, set
 
  47      discrete=False, otherwise leave at its default value of True.
 
  48      """ 
  49      vars = numeric_to_vars(vals, coordnames, indepvar, indepvarname,
 
  50                             indepdomain, all_types_float, discrete) 
  51      return Trajectory(trajname, vars.values(),
 
  52                        parameterized=indepvar is not None)

  53  
 


54 -def convert_ptlabel_events(pts):


55      """Creates an eventTimes-like dictionary from a pointset's labels.
 
  56      (Event X time recorded in a label is recorded as "Event:X")
 
  57      """ 
  58      ev_labels = [] 
  59      for l in pts.labels.getLabels(): 
  60          if 'Event:' in l: 
  61              ev_labels.append(l[6:]) 
  62      event_times = {} 
  63      for l in ev_labels: 
  64          ts = [] 
  65          ix_dict = pts.labels.by_label['Event:'+l] 
  66          # don't use tdict in labels in case indepvararray has been re-scaled
 
  67          for ix in ix_dict.keys(): 
  68              ts.append(pts.indepvararray[ix]) 
  69          ts.sort() 
  70          event_times[l] = ts 
  71      return event_times

  72  
 


73 -def pointset_to_traj(pts):


74      """Convert a pointset into a trajectory using linear interpolation, retaining
 
  75      parameterization by an independent variable if present.""" 
  76      if isparameterized(pts): 
  77          return numeric_to_traj(pts.coordarray, pts.name, pts.coordnames, pts.indepvararray,
 
  78                                 pts.indepvarname, discrete=False) 
  79      else: 
  80          return numeric_to_traj(pts.coordarray, pts.name, pts.coordnames, discrete=False)

  81  
 
  82  
 


83 -class Trajectory(object):


84      """Parameterized and non-parameterized trajectory class.
 
  85      vals must be a sequence of variable objects.
 
  86  
 
  87      Non-parameterized objects are created by implicit function-defined
 
  88      generators, e.g. ImplicitFnGen.
 
  89      """ 
  90  
 


91 -    def __init__(self, name, vals, coordnames=None, modelNames=None,
 
  92                   timeInterval=None, modelEventStructs=None,
 
  93                   eventTimes=None, events=None,
 
  94                   FScompatibleNames=None, FScompatibleNamesInv=None,
 
  95                   abseps=None, globalt0=0, checklevel=0, norm=2,
 
  96                   parameterized=True):


97          if isinstance(name, str): 
  98              self.name = name 
  99          else: 
 100              raise TypeError("name argument must be a string") 
 101          varlist = vals 
 102          if isinstance(varlist, Variable): 
 103              # then a singleton was passed
 
 104              varlist = [varlist] 
 105          elif isinstance(varlist, _seq_types): 
 106              if not isinstance(varlist[0], Variable): 
 107                  raise TypeError("vals argument must contain Variable objects") 
 108          else: 
 109              raise TypeError("vals argument must contain Variable objects") 
 110          if coordnames is None: 
 111              self.coordnames = [v.name for v in varlist] 
 112          else: 
 113              # unlikely case: filter which variables are used
 
 114              # (because of common API with HybridTrajectory)
 
 115              self.coordnames = copy.copy(coordnames) 
 116              varlist_new = [(v.name, v) for v in varlist] 
 117              varlist_new.sort()  # sort by the names 
 118              varlist = [v for (vname, v) in varlist_new if vname in self.coordnames] 
 119          if not isUniqueSeq(self.coordnames): 
 120              raise ValueError("Coordinate names must be unique") 
 121          self.coordnames.sort() 
 122          if abseps is not None: 
 123              for v in varlist: 
 124                  try: 
 125                      v.indepdomain._abseps = abseps 
 126                  except AttributeError: 
 127                      # domain is just an array
 
 128                      pass 
 129                  try: 
 130                      v.depdomain._abseps = abseps 
 131                  except AttributeError: 
 132                      # domain is just an array
 
 133                      pass 
 134                  try: 
 135                      v.trajirange._abseps = abseps 
 136                  except AttributeError: 
 137                      # domain is just an array
 
 138                      pass 
 139                  try: 
 140                      v.trajdrange._abseps = abseps 
 141                  except AttributeError: 
 142                      # domain is just an array
 
 143                      pass 
 144          # non-parameterized trajectory?
 
 145          indepvarname = varlist[0].indepvarname 
 146          if indepvarname in self.coordnames or not parameterized: 
 147              self._parameterized = False 
 148          else: 
 149              self._parameterized = True 
 150          indepvartype = varlist[0].indepvartype 
 151          if not all([compareNumTypes(v.coordtype, float) for v in varlist]): 
 152              coordtype = float 
 153          else: 
 154              coordtype = varlist[0].coordtype 
 155          self.depdomain = {} 
 156          if varlist[0].trajirange is None: 
 157              indepdomain = varlist[0].indepdomain 
 158              self.depdomain[varlist[0].coordname] = varlist[0].depdomain 
 159          else: 
 160              indepdomain = varlist[0].trajirange 
 161              self.depdomain[varlist[0].coordname] = varlist[0].trajdrange 
 162          for v in varlist[1:]: 
 163              if isinstance(indepdomain, ndarray): 
 164                  if v.trajirange is None: 
 165                      if all(v.indepdomain != indepdomain): 
 166                          raise ValueError("Some variables in varlist argument "
 
 167                                  "have different independent variable domains") 
 168                  else: 
 169                      if all(v.trajirange != indepdomain): 
 170                          raise ValueError("Some variables in varlist argument "
 
 171                                  "have different independent variable domains") 
 172              else: 
 173                  if v.trajirange is None: 
 174                      if v.indepdomain != indepdomain: 
 175                          raise ValueError("Some variables in varlist argument "
 
 176                                  "have different independent variable domains") 
 177                  else: 
 178                      if v.trajirange != indepdomain: 
 179                          raise ValueError("Some variables in varlist argument "
 
 180                                  "have different independent variable domains") 
 181              if v.indepvarname != indepvarname: 
 182                  raise ValueError("Some variables in varlist "
 
 183                    "argument have different independent variable names") 
 184              if v.indepvartype != indepvartype: 
 185                  raise ValueError("Some variables in varlist "
 
 186                    "argument have different independent "
 
 187                    "variable types") 
 188              if v.trajdrange is None: 
 189                  self.depdomain[v.coordname] = v.depdomain 
 190              else: 
 191                  self.depdomain[v.coordname] = v.trajdrange 
 192          self.indepvarname = indepvarname 
 193          self.indepvartype = indepvartype 
 194          # self.indepdomain is not checked when traj called, it's just for info
 
 195          self.indepdomain = indepdomain 
 196          self.coordtype = coordtype 
 197          self.variables = {} 
 198          for v in varlist: 
 199              assert isinstance(v, Variable) 
 200              assert v.defined, ("Variables must be defined before building "
 
 201                                 "a Trajectory object") 
 202              self.variables[v.name] = v 
 203          self.dimension = len(varlist) 
 204          self._name_ix_map = invertMap(self.coordnames) 
 205          self.globalt0 = globalt0 
 206          self.checklevel = checklevel   # unused 
 207          # order for norm of points within trajectory
 
 208          self._normord = norm 
 209          # funcspec compatible names especially used when part of a large
 
 210          # model created using hierarchical object names
 
 211          if FScompatibleNames is None: 
 212              self._FScompatibleNames = symbolMapClass() 
 213          else: 
 214              self._FScompatibleNames = FScompatibleNames 
 215          if FScompatibleNamesInv is None: 
 216              if FScompatibleNames is not None: 
 217                  self._FScompatibleNamesInv = FScompatibleNames.inverse() 
 218              else: 
 219                  self._FScompatibleNamesInv = symbolMapClass() 
 220          else: 
 221              self._FScompatibleNamesInv = FScompatibleNamesInv 
 222          # used by Generators that leave their mark here (for reference by
 
 223          # models in which these are embedded, and to be consistent with
 
 224          # HybridTraj API)
 
 225          self.modelNames = modelNames 
 226          if modelEventStructs is None: 
 227              self.modelEventStructs = None 
 228          else: 
 229              self.modelEventStructs = copy.copy(modelEventStructs) 
 230          if events is None: 
 231              self.events = {} 
 232          else: 
 233              self.events = copy.copy(events) 
 234          if eventTimes is None: 
 235              self._createEventTimes() 
 236          else: 
 237              self.eventTimes = eventTimes 
 238          self.timePartitions = [(self.indepdomain, self.globalt0, checklevel)]

 239  
 


240 -    def _createEventTimes(self):


241          eventTimes = {} 
 242          # if events was provided then fill in with that -- most
 
 243          # commonly used by Generators that only pass events object
 
 244          for evname, evpts in self.events.iteritems(): 
 245              if evpts is None: 
 246                  eventTimes[evname] = [] 
 247              else: 
 248                  val = mapNames(self._FScompatibleNamesInv, evpts) 
 249                  if evname in eventTimes: 
 250                      eventTimes[evname].extend(val.indepvararray.tolist()) 
 251                  else: 
 252                      eventTimes[evname] = val.indepvararray.tolist() 
 253          self.eventTimes = eventTimes

 254  
 


255 -    def delete_variables(self, coords):


256          """coords is a list of coordinate names to remove""" 
 257          assert alltrue([c in self.variables for c in coords]), \
 
 258                 "Variable name %s doesn't exist"%c 
 259          assert len(coords) < self.dimension, "Cannot delete every variable!" 
 260          self.coordnames = remain(self.coordnames, coords) 
 261          self._name_ix_map = invertMap(self.coordnames) 
 262          self.dimension -= len(coords) 
 263          for c in coords: 
 264              del self.variables[c] 
 265              del self.depdomain[c]

 266  
 


267 -    def mapNames(self, themap):


268          """themap is a symbolMapClass mapping object for remapping coordinate names
 
 269          """ 
 270          new_coordnames = array(themap(self.coordnames)).tolist() 
 271          assert isUniqueSeq(new_coordnames), 'Coordinate names must be unique' 
 272          new_coordnames.sort() 
 273          if self._parameterized: 
 274              assert self.indepvarname not in new_coordnames, \
 
 275                     'Coordinate names must not overlap with independent variable' 
 276          for c in self.coordnames: 
 277              self.variables[c].name = themap(c) 
 278          self.variables = themap(self.variables) 
 279          self.depdomain = themap(self.depdomain) 
 280          self._FScompatibleNames = themap(self._FScompatibleNames) 
 281          self._FScompatibleNamesInv = self._FScompatibleNames.inverse() 
 282          self.coordnames = new_coordnames 
 283          self._name_ix_map = invertMap(self.coordnames)

 284  
 
 285  
 


286 -    def truncate_to_idx(self, idx):


287          """Truncate trajectory according to a last coordinate specified by idx
 
 288          argument, provided trajectory is defined by an underlying mesh.""" 
 289          t_vals = None 
 290          for vname, v in self.variables.iteritems(): 
 291              # vmesh is an array with two rows, the first = indep var, the second = dep var
 
 292              vmesh = v.underlyingMesh() 
 293              if vmesh is not None: 
 294                  # adjust mesh for this variable
 
 295                  if t_vals is None: 
 296                      t_vals = vmesh[0] 
 297                      if idx > len(t_vals): 
 298                          raise ValueError("Index out of range for truncation of variable"
 
 299                                   " %s in trajectory %s"%(vname, self.name)) 
 300                  v.truncate_to_idx(idx) 
 301                  # adjust self.depdomain if it has changed
 
 302                  if v.trajdrange is None: 
 303                      self.depdomain[v.coordname] = v.depdomain 
 304                  else: 
 305                      self.depdomain[v.coordname] = v.trajdrange 
 306          if self._parameterized: 
 307              new_t_end = t_vals[idx] 
 308              # adjust own independent variable
 
 309              if self.globalt0 > new_t_end: 
 310                  self.globalt0 = new_t_end 
 311              self.indepdomain.set([self.indepdomain[0], new_t_end])

 312  
 


313 -    def truncate_to_indepvar(self, t):


314          """Truncate trajectory according to an independent variable value given
 
 315          by t argument, provided trajectory is defined as parameterized.""" 
 316          #assert self._parameterized, "Only call parameterized trajectories"
 
 317          #if self.globalt0 > t:
 
 318          #    self.globalt0 = t
 
 319          raise NotImplementedError("This feature is not yet implemented")

 320  
 
 321  
 


322 -    def __call__(self, t, coords=None, checklevel=None,
 
 323                   asGlobalTime=False, asmap=False):


324          """Evaluate a parameterized trajectory at given independent
 
 325          variable value(s), interpreted as local times by default, unless
 
 326          asGlobalTime==True (default is False, unlike with sample method).
 
 327  
 
 328          asmap option allows continuous trajectory to be called as a map, with
 
 329          t = 0 or 1 to return the two endpoints. Result includes actual
 
 330          time values (in global time if asGlobalTime==True).
 
 331          """ 
 332          assert self._parameterized, "Only call parameterized trajectories" 
 333          if checklevel is None: 
 334              checklevel = self.checklevel 
 335          if coords is None: 
 336              coordlist = self.coordnames 
 337          elif isinstance(coords, int): 
 338              coordlist = [self.coordnames[coords]] 
 339          elif isinstance(coords, str): 
 340              coordlist = [coords] 
 341          elif isinstance(coords, _seq_types): 
 342              if all([isinstance(c, str) for c in coords]): 
 343                  coordlist = [v for v in self.coordnames if v in coords] 
 344                  if asmap: 
 345                      # allow independent variable name to be listed now
 
 346                      test_names = self.coordnames + [self.indepvarname] 
 347                  else: 
 348                      test_names = copy.copy(self.coordnames) 
 349                  if remain(coords, test_names) != []: 
 350                      print "Valid coordinate names:", self.coordnames 
 351                      raise ValueError("Invalid coordinate names passed") 
 352              elif any([isinstance(c, str) for c in coords]): 
 353                  raise TypeError("Cannot mix string and numeric values in "
 
 354                                    "coords") 
 355              else: 
 356                  # numeric list assumed
 
 357                  coordlist = [v for v in self.coordnames if v in self._name_ix_map] 
 358          else: 
 359              raise TypeError("Invalid type for coords argument") 
 360          if asmap: 
 361              # assumes t will be 0, 1 or list of these only (self.indepdomain
 
 362              # will complain otherwise).
 
 363              # checklevel is not needed for this usage
 
 364              checklevel = 0 
 365              if isinstance(t, _seq_types): 
 366                  for tval in t: 
 367                      # convert to continuous times
 
 368                      t = [self.indepdomain[tval] for tval in t] 
 369              else: 
 370                  # convert to continuous time
 
 371                  t = self.indepdomain[t] 
 372          if isinstance(t, _seq_types): 
 373              if len(t) == 0: 
 374                  raise ValueError("No independent variable value") 
 375              # ensure is an array so that we can subtract globalt0 from all
 
 376              if asGlobalTime and not asmap: 
 377                  indepvals = array(t) - self.globalt0 
 378              else: 
 379                  # variable object will accept any sequence type
 
 380                  indepvals = t 
 381              try: 
 382                  vals = [v(indepvals, checklevel) \
 
 383                          for v in [self.variables[vn] for vn in coordlist]] 
 384              except: 
 385                  if checklevel > 1: 
 386                      print "\nProblem calling with coords:", coordlist 
 387                      print "Indepdendent variable values:", indepvals 
 388                      print "Containment:", self.indepdomain.contains(t) 
 389                      try: 
 390                          print self.variables[coordlist[0]].indepdomain.get() 
 391                          print self.variables[coordlist[0]].depdomain.get() 
 392                      except AttributeError: 
 393                          # domains may be discrete
 
 394                          print "Discrete variable domain and/or range" 
 395  #                print self.variables[coordlist[0]].initialconditions
 
 396                  raise 
 397  #                raise ValueError("Problem calling at these independent variable values")
 
 398              if asmap: 
 399                  if asGlobalTime: 
 400                      vals.append(array(t)) 
 401                  else: 
 402                      # ensure is an array so that we can subtract globalt0 from all
 
 403                      # return t vals in global time coords
 
 404                      # (internally-generated local t vals)
 
 405                      vals.append(array(t) + self.globalt0) 
 406                  coordlist.append(self.indepvarname) 
 407  ##            if len(coordlist) == 1:
 
 408  ##                return array(vals[0], self.coordtype)
 
 409  ##            else:
 
 410              #if asGlobalTime:
 
 411              #    offset = 0
 
 412              #else:
 
 413              offset = self.globalt0 
 414              return self._FScompatibleNamesInv(
 
 415                 Pointset({'coordarray': vals,
 
 416                           'coordnames': coordlist,
 
 417                           'coordtype': self.coordtype,
 
 418                           'indepvararray': array(indepvals)+offset,
 
 419                           'norm': self._normord,
 
 420                           'name': self.name + "_sample"})) 
 421          else: 
 422              if asGlobalTime and not asmap: 
 423                  t = t - self.globalt0 
 424              try: 
 425                  varvals = [v(t, checklevel) for v in \
 
 426                                   [self.variables[vn] for vn in coordlist]] 
 427              except: 
 428                  if checklevel > 1: 
 429                      print "\nProblem calling with coords:", coordlist 
 430                      print "Indepdendent variable values:", t 
 431                      print "Containment:", self.indepdomain.contains(t) 
 432                      try: 
 433                          print self.variables[coordlist[0]].indepdomain.get() 
 434                          print self.variables[coordlist[0]].depdomain.get() 
 435                      except AttributeError: 
 436                          # domains may be discrete
 
 437                          print "Discrete variable domain and/or range" 
 438                  raise 
 439              if asmap: 
 440                  coordlist.append(self.indepvarname) 
 441                  if asGlobalTime: 
 442                      varvals.append(t) 
 443                  else: 
 444                      # return t val in global time, so have to
 
 445                      # add globalt0 to the internally-generated local t val
 
 446                      varvals.append(t + self.globalt0) 
 447  ##            if len(coordlist) == 1:
 
 448  ##                # covers asmap == False case
 
 449  ##                return self.variables[coordlist[0]](t, checklevel)
 
 450  ##            else:
 
 451              #if asGlobalTime:
 
 452              #    offset = 0
 
 453              #else:
 
 454              offset = self.globalt0 
 455              return self._FScompatibleNamesInv(
 
 456                  Point({'coordarray': varvals,
 
 457                         'coordnames': coordlist,
 
 458                         'coordtype': self.coordtype,
 
 459                         'indepvararray': array(t)+offset,
 
 460                         'norm': self._normord}))

 461  
 
 462  
 


463 -    def underlyingMesh(self, coords=None, FScompat=True):


464          """Return a dictionary of the underlying independent variables` meshes,
 
 465          where they exist. (Dictionary contains references to the meshes,
 
 466          not copies of the meshes.) Always returns data in local time frame.
 
 467  
 
 468          FScompat option selects whether to use FuncSpec compatible
 
 469          naming for variables (mainly for internal use).""" 
 470          # contrast to code to pull out both dependent and independent
 
 471          # variables' data points in Variable class' getDataPoints
 
 472          if coords is None: 
 473              coords = self.coordnames 
 474          if not isinstance(coords, list): 
 475              raise TypeError('coords argument must be a list') 
 476          meshdict = {} 
 477          if FScompat: 
 478              cs = self._FScompatibleNames(coords) 
 479          else: 
 480              cs = coords 
 481          for varname in cs: 
 482              meshdict[varname] = self.variables[varname].underlyingMesh() 
 483          if FScompat: 
 484              return self._FScompatibleNamesInv(meshdict) 
 485          else: 
 486              return meshdict

 487  
 
 488  
 


489 -    def sample(self, coords=None, dt=None, tlo=None, thi=None,
 
 490                     doEvents=True, precise=False, asGlobalTime=True):


491          """Uniformly sample the named trajectory over range indicated.
 
 492          Returns a Pointset.
 
 493  
 
 494          If doEvents=True (default), the event points for the trajectory
 
 495            are included in the output, regardless of the dt sampling rate.
 
 496  
 
 497          The order of variable names given in the 'coords' argument is ignored.
 
 498  
 
 499          precise=True causes the trajectory position to be evaluated precisely
 
 500            at the t values specified, which will invoke slow interpolation
 
 501            (requires dt to be set otherwise an exception will be raised).
 
 502          precise=False (default) causes the nearest underlying mesh positions
 
 503            to be used, if available (otherwise the behaviour is the same as
 
 504            precise=True provided dt has been set, otherwise an exception will be
 
 505            raised).
 
 506  
 
 507          If dt is not given, the underlying time mesh is used, if available.
 
 508          """ 
 509  
 
 510          if coords is None: 
 511              coords_sorted = self.coordnames 
 512          else: 
 513              if not isinstance(coords, list): 
 514                  assert isinstance(coords, str), \
 
 515                         "coords must be a list of strings or a singleton string" 
 516                  coords_sorted = [self._FScompatibleNames(coords)] 
 517              else: 
 518                  coords_sorted = self._FScompatibleNames(coords) 
 519                  coords_sorted.sort() 
 520          # keep these as separate calls in case of singleton domain (won't unpack properly)
 
 521          tlo_base = self.indepdomain.get(0) 
 522          thi_base = self.indepdomain.get(1) 
 523          if tlo is None: 
 524              tlo = tlo_base 
 525          elif asGlobalTime: 
 526              tlo = tlo - self.globalt0 
 527              if tlo < tlo_base: 
 528                  tlo = tlo_base 
 529              if tlo >= thi_base: 
 530                  # message used by HybridTrajectory.sample
 
 531                  raise ValueError("tlo too large") 
 532          elif tlo < tlo_base: 
 533              tlo = tlo_base 
 534          elif tlo >= thi_base: 
 535              # message used by HybridTrajectory.sample
 
 536              raise ValueError("tlo too large") 
 537          if thi is None: 
 538              thi = thi_base 
 539          elif asGlobalTime: 
 540              thi = thi - self.globalt0 
 541              if thi > thi_base: 
 542                  thi = thi_base 
 543              if thi <= tlo_base: 
 544                  # message used by HybridTrajectory.sample
 
 545                  raise ValueError("thi too small") 
 546          elif thi > thi_base: 
 547              thi = thi_base 
 548          elif thi <= tlo_base: 
 549              # message used by HybridTrajectory.sample
 
 550              raise ValueError("thi too small") 
 551          if self.indepdomain.issingleton: 
 552              # res will be an array if coords_sorted is a singleton,
 
 553              # otherwise a pointset
 
 554              res = self.__call__([self.indepdomain.get()], coords_sorted) 
 555              try: 
 556                  res.name = self.name + "_sample" 
 557              except AttributeError: 
 558                  # res is array
 
 559                  pass 
 560              return res 
 561          else: 
 562              assert tlo < thi, 't start point must be less than t endpoint' 
 563              if dt is not None and dt >= abs(thi-tlo): 
 564                  if precise: 
 565                      print "dt = %f for interval [%f,%f]"%(dt,tlo,thi) 
 566                      raise ValueError('dt must be smaller than time interval') 
 567                  else: 
 568                      dt = (thi-tlo)/10. 
 569          if precise: 
 570              if dt is None: 
 571                  raise ValueError("Must specify an explicit dt when precise flag"
 
 572                                   " is set") 
 573              else: 
 574                  tmesh = concatenate((arange(tlo, thi, dt),[thi])) 
 575                  loix = 0 
 576                  hiix = len(tmesh) 
 577                  meshes_ok = True 
 578          else: 
 579              # not precise, so attempt to use underlying mesh for each variable,
 
 580              # if available (always in local timeframe)
 
 581              meshdict = self.underlyingMesh(FScompat=False) 
 582              meshes_ok = False 
 583              if meshdict is not None: 
 584                  if not any([meshdict[v] is None for v in coords_sorted]): 
 585                      # ensure all vars' meshes are identical!
 
 586                      firstvar_tmesh = asarray(meshdict[coords_sorted[0]][0].copy()) 
 587                      meshes_ok = True 
 588                      # explicitly checking that the arrays are the same
 
 589                      # is very slow -- just catch a resulting error later
 
 590              if meshes_ok: 
 591                  loix_a = ( firstvar_tmesh >= tlo ).tolist() 
 592                  try: 
 593                      loix = loix_a.index(1) 
 594                  except ValueError: 
 595                      loix = 0 
 596                  hiix_a = ( firstvar_tmesh > thi ).tolist() 
 597                  try: 
 598                      hiix = hiix_a.index(1) 
 599                  except ValueError: 
 600                      hiix = len(firstvar_tmesh) 
 601                  firstvar_tmesh = firstvar_tmesh[loix:hiix] 
 602                  if dt is None: 
 603                      tmesh = firstvar_tmesh 
 604                  else: 
 605                      # this is just the goal mesh, but with precise=False
 
 606                      # only the closest actual mesh points will be used
 
 607                      tmesh = concatenate((arange(tlo, thi, dt), [thi])) 
 608                      # get closest mesh indices corresponding to tmesh
 
 609                      # times for first var
 
 610                      closest = makeSeqUnique(findClosestArray(firstvar_tmesh,
 
 611                                                         tmesh, dt/2), True) 
 612                      # filter any out-of-range tvals picked
 
 613                      tmesh = array([t for t in firstvar_tmesh[closest] \
 
 614                                     if t >= tlo and t <= thi]) 
 615  
 
 616          if meshes_ok: 
 617              ix_range = arange(loix, hiix) 
 618          else: 
 619              if dt is None: 
 620                  raise PyDSTool_ValueError("Underlying mesh of trajectory is not"
 
 621                               " the same for all variables: dt must "
 
 622                               "be specified in this case") 
 623              else: 
 624                  # tmesh not created
 
 625                  precise = True 
 626                  tmesh = concatenate((arange(tlo, thi, dt), [thi])) 
 627  
 
 628          # check for loss of precision when restoring to global time,
 
 629          # and if present, just filter out "equal" times
 
 630          if asGlobalTime: 
 631              tmesh_glob = tmesh + self.globalt0 
 632              if not isincreasing(tmesh_glob): 
 633                  # don't need tmesh_glob_filtered here
 
 634                  tmesh_glob, good_ixs = unique(tmesh_glob, True) 
 635                  tmesh = tmesh[good_ixs] 
 636                  ix_range = ix_range[good_ixs] 
 637          else: 
 638              tmesh_glob = tmesh 
 639  
 
 640  
 
 641          # Add Pointset labels for events
 
 642          # Work in global time in case of loss of precision in small
 
 643          # time differences between time points and event points
 
 644          evlabels = PointInfo() 
 645          if doEvents: 
 646              eventdata = self.getEventTimes(asGlobalTime=True) 
 647              eventpts = self.getEvents(asGlobalTime=True) 
 648              evtlist = [(t, evname) for (t, evname) in \
 
 649                         orderEventData(eventdata, bytime=True) \
 
 650                         if t >= tmesh_glob[0] - self.indepdomain._abseps and \
 
 651                            t <= tmesh_glob[-1] + self.indepdomain._abseps] 
 652              ev_pts_list = [] 
 653              if evtlist != []: 
 654                  ev_ts = [t for (t, evname) in evtlist] 
 655                  for t, evname in evtlist: 
 656                      # get each state point
 
 657                      tix = eventpts[evname].find(t) 
 658                      ev_pts_list.append(eventpts[evname][tix]) 
 659                  tmesh_glob, ins_ixs, close_ix_dict = insertInOrder( \
 
 660                                                 tmesh_glob,
 
 661                                                 ev_ts, return_ixs=True,
 
 662                                                 abseps=self.indepdomain._abseps) 
 663              else: 
 664                  ins_ixs = [] 
 665              if len(ins_ixs) != len(evtlist): 
 666                  # check to see if events need to be inserted at start
 
 667                  # or end of tmesh (insertInOrder doesn't do that)
 
 668                  # -- it may just be that the event times are already in tmesh
 
 669                  start_ins = [] 
 670                  for t in ev_ts: 
 671                      if t < tmesh_glob[0] - self.indepdomain._abseps: 
 672                          start_ins.append(t) 
 673                      else: 
 674                          break 
 675                  end_ins = [] 
 676                  for t in ev_ts[::-1]: 
 677                      if t - tmesh_glob[-1] > self.indepdomain._abseps: 
 678                          end_ins.append(t) 
 679                      else: 
 680                          break 
 681                  end_ins.reverse() 
 682                  if start_ins != [] or end_ins != []: 
 683                      lastins = len(tmesh_glob) 
 684                      lenstart = len(start_ins) 
 685                      tmesh_glob = concatenate((start_ins, tmesh_glob, end_ins)) 
 686                      ins_ixs = range(lenstart) + [i+lenstart for i in ins_ixs] + \
 
 687                              range(lastins+lenstart, lastins+lenstart+len(end_ins)) 
 688              tmesh_list = tmesh_glob.tolist() 
 689              for i, (t, evname) in enumerate(evtlist): 
 690                  try: 
 691                      ix = ins_ixs[i] 
 692                  except IndexError: 
 693                      # already in tmesh
 
 694                      try: 
 695                          # t may only match up to rounding error
 
 696                          ix = close_ix_dict[t] 
 697                      except KeyError: 
 698                          # t will match exactly
 
 699                          ix = tmesh_list.index(t) 
 700                  if asGlobalTime: 
 701                      evlabels.update(ix, 'Event:'+evname, {'t': t}) 
 702                  else: 
 703                      evlabels.update(ix, 'Event:'+evname, {'t': t-self.globalt0}) 
 704          else: 
 705              eventdata = None 
 706  
 
 707          if asGlobalTime: 
 708              tmesh = tmesh_glob - self.globalt0 
 709          else: 
 710              tmesh = tmesh_glob 
 711  
 
 712          if len(tmesh) > 0: 
 713              if dt is None: 
 714                  coorddict = dict(zip(coords_sorted, [m[1][ix_range] for m in \
 
 715                                   sortedDictValues(meshdict,
 
 716                                                  onlykeys=coords_sorted)])) 
 717                  if eventdata: 
 718                      # insert var values at events (SLOW!)
 
 719                      # can only insert to lists, so have to convert coorddict
 
 720                      # arrays to lists first.
 
 721                      for tpos in xrange(len(ins_ixs)): 
 722                          tix = ins_ixs[tpos] 
 723                          t = ev_ts[tpos] 
 724                          x = self._FScompatibleNames(ev_pts_list[tpos]) 
 725                          for v in coords_sorted: 
 726                              try: 
 727                                  coorddict[v].insert(tix, x[v]) 
 728                              except AttributeError: 
 729                                  coorddict[v] = coorddict[v].tolist() 
 730                                  coorddict[v].insert(tix, x[v]) 
 731                  # if asGlobalTime, tmesh already includes
 
 732                  # + self.globalt0 offset to tlo, thi, but must
 
 733                  # return independent var values in global timeframe
 
 734                  if asGlobalTime: 
 735                      # have to watch out for loss of precision here, especially
 
 736                      # due to events very close together
 
 737                      tmesh += self.globalt0 
 738                  return self._FScompatibleNamesInv(
 
 739                          Pointset({'coorddict': coorddict,
 
 740                                    'coordtype': float64,
 
 741                                    'coordnames': coords_sorted,
 
 742                                    'indepvararray': tmesh,
 
 743                                    'indepvarname': self.indepvarname,
 
 744                                    'indepvartype': float64,
 
 745                                    'norm': self._normord,
 
 746                                    'name': self.name + "_sample",
 
 747                                    'labels': evlabels})) 
 748              elif not precise: 
 749                  try: 
 750                      coorddict = {} 
 751                      for v in coords_sorted: 
 752                          dat = self.variables[v].output.datapoints[1] 
 753                          try: 
 754                              coorddict[v] = dat[closest+loix] 
 755                          except TypeError: 
 756                              dat = array(dat) 
 757                              coorddict[v] = dat[closest+loix] 
 758                  except (AttributeError, IndexError): 
 759                      # meshes of variables did not match up, so
 
 760                      # continue to 'else' case, as if precise=True
 
 761                      pass 
 762                  else: 
 763                      if eventdata: 
 764                          # insert var values at events (SLOW!)
 
 765                          # can only insert to lists, so have to convert coorddict
 
 766                          # arrays to lists first.
 
 767                          for tpos in xrange(len(ins_ixs)): 
 768                              tix = ins_ixs[tpos] 
 769                              t = ev_ts[tpos] 
 770                              x = self._FScompatibleNames(ev_pts_list[tpos]) 
 771                              for v in coords_sorted: 
 772                                  try: 
 773                                      coorddict[v].insert(tix, x[v]) 
 774                                  except AttributeError: 
 775                                      coorddict[v] = coorddict[v].tolist() 
 776                                      coorddict[v].insert(tix, x[v]) 
 777                      # if asGlobalTime, tmesh already includes
 
 778                      # self.globalt0 offset to tlo, thi, but must
 
 779                      # return independent var values in global timeframe
 
 780                      if asGlobalTime: 
 781                          tmesh += self.globalt0 
 782                      return self._FScompatibleNamesInv(
 
 783                          Pointset({'coorddict': coorddict,
 
 784                                     'coordtype': float64,
 
 785                                     'coordnames': coords_sorted,
 
 786                                     'indepvararray': tmesh,
 
 787                                     'indepvarname': self.indepvarname,
 
 788                                     'indepvartype': float64,
 
 789                                     'norm': self._normord,
 
 790                                     'name': self.name + "_sample",
 
 791                                     'labels': evlabels})) 
 792  
 
 793              # else mesh not available for some variables so we have
 
 794              # a mixed-source trajectory, and simple solution is to
 
 795              # treat as if precise==True
 
 796              # Cases: either precise == True or could not extract
 
 797              # underlying meshes for some variables, or the meshes were not
 
 798              # identical.
 
 799              # call self with asGlobalTime=False b/c tmesh already in "local"
 
 800              # time frame, global offset already adjusted for.
 
 801              vals = self(tmesh, coords_sorted, asGlobalTime=False) 
 802              try: 
 803                  # returned pointset
 
 804                  vals = vals.toarray() 
 805              except AttributeError: 
 806                  # was array already
 
 807                  pass 
 808              pset = Pointset({'coordarray': vals,
 
 809                       'coordnames': coords_sorted,
 
 810                       'indepvararray': array(tmesh),   # globalt0 added later
 
 811                       'indepvarname': self.indepvarname,
 
 812                       'name': self.name + "_sample",
 
 813                       'labels': evlabels}) 
 814              # if coords was not sorted, call to self could potentially return
 
 815              # an array in a different order to that of coords.
 
 816              if asGlobalTime and self.globalt0 != 0: 
 817                  pset.indepvararray += self.globalt0 
 818                  pset.makeIxMaps() 
 819              return self._FScompatibleNamesInv(pset) 
 820          else: 
 821              return None

 822  
 
 823  
 


824 -    def __copy__(self):


825          pickledself = pickle.dumps(self) 
 826          c = pickle.loads(pickledself) 
 827  ##        c.indepdomain = copy.copy(self.indepdomain)
 
 828  ##        c.depdomain = copy.copy(self.depdomain)
 
 829  ##        for vname, v in self.variables.iteritems():
 
 830  ##            c.variables[vname] = copy.copy(v)
 
 831          return c

 832  
 
 833  
 


834 -    def __deepcopy__(self, memo=None, _nil=[]):


835          pickledself = pickle.dumps(self) 
 836          return pickle.loads(pickledself)

 837  
 
 838  
 


839 -    def __getstate__(self):


840          d = copy.copy(self.__dict__) 
 841          # remove reference to Cfunc types by converting them to strings
 
 842          d['indepvartype'] = _num_type2name[self.indepvartype] 
 843          d['coordtype'] = _num_type2name[self.coordtype] 
 844          return d

 845  
 
 846  
 


847 -    def __setstate__(self, state):


848          self.__dict__.update(state) 
 849          # reinstate Cfunc types
 
 850          self.indepvartype = _num_name2type[self.indepvartype] 
 851          self.coordtype = _num_name2type[self.coordtype]

 852  
 
 853  
 


854 -    def __del__(self):


855          # necessary for __del__ methods of variables to be accessed
 
 856          for v in self.variables.values(): 
 857              v.__del__()

 858  
 
 859  
 


860 -    def __repr__(self):


861          return self._infostr(verbose=0)

 862  
 
 863  
 
 864      __str__ = __repr__ 
 865  
 
 866  
 


867 -    def _infostr(self, verbose=1):


868          if verbose <= 0: 
 869              outputStr = "Trajectory " + self.name 
 870          elif verbose >= 1: 
 871              outputStr = "Trajectory " + self.name + "\n  of variables: " + \
 
 872                      str(self._FScompatibleNamesInv(self.variables.keys())) 
 873              outputStr += "\n  over domains: " + str(self.depdomain) 
 874              if verbose == 2: 
 875                  outputStr += joinStrs(["\n"+v._infostr(1) for v in \
 
 876                                              self.variables.values()]) 
 877          return outputStr

 878  
 


879 -    def info(self, verboselevel=1):


880          print self._infostr(verboselevel)

 881  
 
 882  
 


883 -    def getEvents(self, evnames=None, asGlobalTime=True):


884          """Returns a pointset of all named events occuring in global time,
 
 885          unless asGlobalTime option set to False (default is True).
 
 886          If no events are named, all are used.
 
 887          """ 
 888          # self.events is a dict of pointsets keyed by event name
 
 889          if evnames is None: 
 890              evnames = self.events.keys() 
 891          if isinstance(evnames, str): 
 892              # singleton
 
 893              assert evnames in self.events, "Invalid event name provided: %s"%evnames 
 894              result = copy.copy(self.events[evnames]) 
 895              if asGlobalTime: 
 896                  try: 
 897                      result.indepvararray += self.globalt0 
 898                  except AttributeError: 
 899                      # empty pointset
 
 900                      pass 
 901          else: 
 902              result = {} 
 903              # assume iterable
 
 904              assert all([ev in self.events for ev in evnames]), \
 
 905                     "Invalid event name(s) provided: %s"%str(evnames) 
 906              for evname in evnames: 
 907                  evptset = self.events[evname] 
 908                  result[evname] = copy.copy(evptset) 
 909                  if asGlobalTime and self.globalt0 != 0: 
 910                      try: 
 911                          result[evname].indepvararray += self.globalt0 
 912                      except AttributeError: 
 913                          # empty pointset
 
 914                          pass 
 915          return result

 916  
 
 917  
 


918 -    def getEventTimes(self, evnames=None, asGlobalTime=True):


919          """Returns a list of times at which the named events occurred in global
 
 920          time, unless asGlobalTime option set to False (default is True).
 
 921          If no events are named, all are used.
 
 922          """ 
 923          result = {} 
 924          if evnames is None: 
 925              evnames = self.events.keys() 
 926          # self.eventTimes is a dict of lists keyed by event name
 
 927          if isinstance(evnames, str): 
 928              # singleton
 
 929              assert evnames in self.events, "Invalid event name provided: %s"%evnames 
 930              if asGlobalTime: 
 931                  result = list(array(self.eventTimes[evnames]) + self.globalt0) 
 932              else: 
 933                  result = self.eventTimes[evnames] 
 934          else: 
 935              if asGlobalTime: 
 936                  t_offset = self.globalt0 
 937              else: 
 938                  t_offset = 0 
 939              # assume iterable
 
 940              assert all([ev in self.events for ev in evnames]), \
 
 941                     "Invalid event name(s) provided %s"%str(evnames) 
 942              for evname in evnames: 
 943                  try: 
 944                      evtimes = self.eventTimes[evname] 
 945                  except KeyError: 
 946                      pass 
 947                  else: 
 948                      result[evname] = list(array(evtimes) + t_offset) 
 949          return result

 950  
 
 951  
 
 952  
 
 953  
 


954 -class HybridTrajectory(Trajectory):


955      """Hybrid, parameterized, trajectory class.
 
 956      Mimics API of a non-hybrid Trajectory.
 
 957  
 
 958      vals must be a sequence of trajectory segments.
 
 959      coords is optional (restricts variables to store).
 
 960      """ 


961 -    def __init__(self, name, vals, coordnames=None, modelNames=None,
 
 962                   timeInterval=None, modelEventStructs=None,
 
 963                   eventTimes=None, events=None,
 
 964                   FScompatibleNames=None, FScompatibleNamesInv=None,
 
 965                   abseps=None, globalt0=0, checklevel=0, norm=2,
 
 966                   timePartitions=None):


967          if isinstance(name, str): 
 968              self.name = name 
 969          else: 
 970              raise TypeError("name argument must be a string") 
 971          trajSeq = vals 
 972          self.indepvarname = trajSeq[0].indepvarname 
 973          if coordnames is None: 
 974              # initial value for coordnames only
 
 975              self.coordnames = trajSeq[0].coordnames 
 976              # choose coordnames to be all common variable names
 
 977              for traj in trajSeq: 
 978                  self.coordnames = intersect(self.coordnames, traj.coordnames) 
 979                  assert traj.indepvarname == self.indepvarname, \
 
 980                           "Inconsistent independent variable names" 
 981          else: 
 982              self.coordnames = copy.copy(coordnames) 
 983              for traj in trajSeq: 
 984                  assert traj.indepvarname == self.indepvarname, \
 
 985                           "Inconsistent independent variable names" 
 986              if not isUniqueSeq(self.coordnames): 
 987                  raise ValueError("Coordinate names must be unique") 
 988          self.coordnames.sort() 
 989          # don't enforce self.indepvartype == trajSeq[i].indepvartype
 
 990          # for when hybrid trajectories can consist of map and non-map parts
 
 991          # (some time points will simply not exist in those cases)
 
 992          self.indepvartype = trajSeq[0].indepdomain.type 
 993          self.coordtype = float    # may have to change this 
 994          if timeInterval is None: 
 995              # extract from trajSeq
 
 996              tlo = trajSeq[0].indepdomain[0] 
 997              thi = trajSeq[-1].indepdomain[1] 
 998              timeInterval = Interval(self.indepvarname, self.indepvartype,
 
 999                                           [tlo, thi], abseps=self._abseps) 
1000          # partitions are (interval, globalt0, checklevel) triples
 
1001          self.timePartitions = timePartitions 
1002          # sequence (list) of other Trajectory or HybridTrajectory objects
 
1003          self.trajSeq = trajSeq 
1004          # list of models generating each traj in trajSeq
 
1005          self.modelNames = modelNames 
1006          self.modelEventStructs = modelEventStructs 
1007          if events is None: 
1008              self.events = {} 
1009          else: 
1010              self.events = events 
1011          if eventTimes is None: 
1012              self.eventTimes = {} 
1013          else: 
1014              self.eventTimes = eventTimes 
1015          if FScompatibleNames is None: 
1016              self._FScompatibleNames = symbolMapClass() 
1017          else: 
1018              self._FScompatibleNames = FScompatibleNames 
1019          if FScompatibleNamesInv is None: 
1020              self._FScompatibleNamesInv = symbolMapClass() 
1021          else: 
1022              self._FScompatibleNamesInv = FScompatibleNamesInv 
1023          # mimic regular variables attribute of non-hybrid Trajectory
 
1024          self.variables = {} 
1025          self.depdomain = {} 
1026          for ov in self.coordnames: 
1027              self.variables[ov] = HybridVariable(self, ov,
 
1028                                              timeInterval, abseps=abseps) 
1029              self.depdomain[ov] = Interval(ov, float, [-Inf, Inf],
 
1030                                              abseps=abseps) 
1031          self.globalt0 = globalt0 
1032          self.indepdomain = timeInterval 
1033          self._parameterized = True 
1034          self.dimension = len(self.coordnames) 
1035          self._name_ix_map = invertMap(self.coordnames) 
1036          self.checklevel = checklevel    # unused 
1037          # order for norm of points within trajectory
 
1038          self._normord = norm

1039  
 


1040 -    def underlyingMesh(self, coords=None, FScompat=True):


1041          """Return a dictionary of the underlying independent variables` meshes,
 
1042          where they exist. (Dictionary contains references to the meshes,
 
1043          not copies of the meshes.) Always returns data in local time frame.
 
1044  
 
1045          FScompat option selects whether to use FuncSpec compatible
 
1046          naming for variables (mainly for internal use).""" 
1047          # contrast to code to pull out both dependent and independent
 
1048          # variables' data points in Variable class' getDataPoints
 
1049          if coords is None: 
1050              coords = self.coordnames 
1051          if not isinstance(coords, list): 
1052              raise TypeError('coords argument must be a list') 
1053          meshdict = {} 
1054          if FScompat: 
1055              cs = self._FScompatibleNames(coords) 
1056          else: 
1057              cs = coords 
1058          for varname in cs: 
1059              try: 
1060                  meshdict[varname] = self.variables[varname].underlyingMesh() 
1061              except AttributeError: 
1062                  meshdict[varname] = None 
1063          if FScompat: 
1064              return self._FScompatibleNamesInv(meshdict) 
1065          else: 
1066              return meshdict

1067  
 


1068 -    def showRegimes(self):


1069          parts = [p[1] for p in self.timePartitions] 
1070          for g, p in zip(self.modelNames, parts): 
1071              print "Regime: %s, from t = %.5f"%(g,p)

1072  
 


1073 -    def info(self):


1074          return info(self, "Hybrid Trajectory")

1075  
 
1076  
 


1077 -    def __call__(self, t, coords=None, checklevel=0,
 
1078                   asGlobalTime=False, asmap=False):


1079          """Evaluate a parameterized hybrid trajectory at given independent
 
1080          variable value(s), interpreted as global times if optional
 
1081          asGlobalTime==True (default is False).
 
1082  
 
1083          asmap option allows the trajectory to be called as a map, with
 
1084          t = integer values to return the values at successive hybrid
 
1085          event times. Result includes actual time values
 
1086          (in global time if asGlobalTime==True).
 
1087          """ 
1088          if checklevel is None: 
1089              checklevel = self.checklevel 
1090          if coords is None: 
1091              coords = copy.copy(self.coordnames) 
1092          else: 
1093              if isinstance(coords, str): 
1094                  coords = [self._FScompatibleNames(coords)] 
1095              elif isinstance(coords, list): 
1096                  coords = self._FScompatibleNames(coords) 
1097              if not [varname in self.coordnames for varname in coords]: 
1098                  raise ValueError('one or more variable names not in observable'
 
1099                               ' variable list') 
1100          if asGlobalTime and not asmap: 
1101              if isinstance(t, _seq_types): 
1102                  # ensure is an array so that we can subtract globalt0 from all
 
1103                  t = array(t) - self.globalt0 
1104              else: 
1105                  t = t - self.globalt0 
1106          time_interval = self.indepdomain 
1107          # timePartitions are (interval, globalt0, checklevel) triples
 
1108          time_partitions = self.timePartitions 
1109          trajseq = self.trajSeq 
1110          if asmap: 
1111              # hybrid system treated as mapping -- include real time value
 
1112              # in returned point / pointset
 
1113              coords.append(self.indepvarname) 
1114              num_parts = len(time_partitions) 
1115              if isinstance(t, _seq_types): 
1116                  dim = len(coords) 
1117                  result = [] 
1118                  for i in range(dim): 
1119                      result.append([]) 
1120                  for tval in t: 
1121                      if not isinstance(tval, _int_types): 
1122                          raise TypeError("time values must be of integer type "
 
1123                           "when treating system as a mapping") 
1124                      if tval < num_parts: 
1125                          # return first point of partition t
 
1126                          part_interval = time_partitions[tval][0] 
1127                          callvals = trajseq[tval](0, coords,
 
1128                                          asGlobalTime=False, asmap=True) 
1129                          if dim == 1: 
1130                              # callvals is just a float
 
1131                              result[0].append(callvals) 
1132                          else: 
1133                              for i in range(dim): 
1134                                  result[i].append(callvals(coords[i])) 
1135                          # adjust time
 
1136                          result[dim-1][-1] += part_interval[0] 
1137                      elif tval == num_parts: 
1138                          # return final point of final partition
 
1139                          part_interval = time_partitions[num_parts-1][0] 
1140                          callvals = trajseq[num_parts-1](1, coords,
 
1141                                          asGlobalTime=False, asmap=True) 
1142                          if dim == 1: 
1143                              # callvals is just a float
 
1144                              result[0].append(callvals) 
1145                          else: 
1146                              for i in range(dim): 
1147                                  result[i].append(callvals(coords[i])) 
1148                          # adjust time
 
1149                          result[dim-1][-1] += part_interval[1] 
1150                      else: 
1151                          raise ValueError("time values not in valid "
 
1152                                             "partition range") 
1153                  return Pointset({'coordarray': array(result),
 
1154                       'coordnames': self._FScompatibleNamesInv(coords),
 
1155                       'indepvartype': int32,
 
1156                       'indepvararray': t,
 
1157                       'indepvarname': 'event',
 
1158                       'norm': self._normord
 
1159                       }) 
1160              else: 
1161                  if not isinstance(t, int): 
1162                      raise TypeError("time value must be an integer"
 
1163                                      " when treating system as a mapping") 
1164                  if t not in range(num_parts+1): 
1165                      raise PyDSTool_BoundsError("time value not in valid"
 
1166                                                   " partition range") 
1167                  if t < num_parts: 
1168                      # return first point of partition t
 
1169                      part_interval = time_partitions[t][0] 
1170                      result = trajseq[t](0, coords,
 
1171                              asGlobalTime=False, asmap=True).toarray().tolist() 
1172                      # adjust time (final entry of result)
 
1173                      result[-1] += part_interval[0] 
1174                  else: 
1175                      # return final point of final partition
 
1176                      part_interval = time_partitions[num_parts-1][0] 
1177                      result = trajseq[num_parts-1](1, coords,
 
1178                              asGlobalTime=False, asmap=True).toarray().tolist() 
1179                      # adjust time (final entry of result)
 
1180                      result[-1] += part_interval[1] 
1181                  return Point({'coordarray': array(result),
 
1182                                'coordnames': \
 
1183                                    self._FScompatibleNamesInv(coords)+[self.indepvarname],
 
1184                                'norm': self._normord
 
1185                               }) 
1186          else: 
1187              # hybrid system treated as curve
 
1188              if not isinstance(t, _seq_types): 
1189                  t = [t] 
1190              retvals = [] 
1191              # lastpart_pos optimizes the re-searching of the time
 
1192              # partitions for long ranges of t
 
1193              ## CURRENTLY UNUSED
 
1194              ##lastpart_pos = 0
 
1195              tix = 0 
1196              while tix < len(t): 
1197                  # list of t vals in a partition, to allow a
 
1198                  # group-call to the trajectory for that partition
 
1199                  trel_part = [] 
1200                  tval = t[tix] 
1201                  if time_interval.contains(tval) is notcontained: 
1202                      print "\n** Debugging info for Hybrid Traj %s: t value, interval, tolerance ="%self.name 
1203                      print tval, time_interval.get(), time_interval._abseps 
1204                      raise PyDSTool_BoundsError('time value outside of '
 
1205                          'trajectory`s time interval '
 
1206                          'of validity (if checklevel was >=2 then endpoints '
 
1207                          'were not included in trajectory)') 
1208                  trajseq_pos = 0 
1209                  tfound = False 
1210                  for (part_interval, gt0, cl) in time_partitions: 
1211                      # trelative
 
1212                      trelative = tval - gt0   # a source of rounding error 
1213                      contresult = part_interval.contains(trelative) 
1214                      if contresult is contained: 
1215                          # find more t vals in this partition, if any
 
1216                          trel_part.append(trelative) 
1217                          part_done = False 
1218                          while not part_done: 
1219                              tix += 1 
1220                              if tix >= len(t): 
1221                                  part_done = True 
1222                                  continue  # while loop 
1223                              trel = t[tix] - gt0   # a source of rounding error 
1224                              contresult_sub = part_interval.contains(trel) 
1225                              if contresult_sub is contained: 
1226                                  trel_part.append(trel) 
1227                              elif contresult_sub is uncertain: 
1228                                  try: 
1229                                      dummy = trajseq[trajseq_pos](trel,
 
1230                                                          asGlobalTime=False,
 
1231                                                          checklevel=checklevel) 
1232                                  except (ValueError, PyDSTool_BoundsError): 
1233                                      # traj segment didn't accept this t value
 
1234                                      # exit if statement and increment trajseq_pos
 
1235                                      call_ok = False 
1236                                  else: 
1237                                      call_ok = True 
1238                                  if call_ok: 
1239                                      trel_part.append(trel) 
1240                                  else: 
1241                                      part_done = True 
1242                              else: 
1243                                  part_done = True 
1244                          tfound = True 
1245                          break  # for loop 
1246                      elif contresult is uncertain: 
1247                          # first verify that this partition's trajectory
 
1248                          # segment will accept this value ... then,
 
1249                          # find more t vals in this partition, if any
 
1250                          try: 
1251                              dummy = trajseq[trajseq_pos](trelative,
 
1252                                                           asGlobalTime=False,
 
1253                                                           checklevel=checklevel) 
1254                          except (ValueError, PyDSTool_BoundsError): 
1255                              # traj segment didn't accept this t value
 
1256                              # exit if statement and increment trajseq_pos
 
1257                              call_ok = False 
1258                          else: 
1259                              call_ok = True 
1260                          if call_ok: 
1261                              trel_part.append(trelative) 
1262                              part_done = False 
1263                              while not part_done: 
1264                                  tix += 1 
1265                                  if tix >= len(t): 
1266                                      part_done = True 
1267                                      continue  # while loop 
1268                                  trel = t[tix] - gt0   # a source of rounding error 
1269                                  contresult_sub = part_interval.contains(trel) 
1270                                  if contresult_sub is contained: 
1271                                      trel_part.append(trel) 
1272                                  elif contresult_sub is uncertain: 
1273                                      try: 
1274                                          dummy = trajseq[trajseq_pos](trel,
 
1275                                                              asGlobalTime=False,
 
1276                                                              checklevel=checklevel) 
1277                                      except (ValueError, PyDSTool_BoundsError): 
1278                                          # traj segment didn't accept this t value
 
1279                                          # exit if statement and increment trajseq_pos
 
1280                                          call_ok = False 
1281                                      else: 
1282                                          call_ok = True 
1283                                      if call_ok: 
1284                                          trel_part.append(trel) 
1285                                      else: 
1286                                          part_done = True 
1287                                  else: 
1288                                      part_done = True 
1289                              tfound = True 
1290                              break  # for loop 
1291                      trajseq_pos += 1 
1292                  if tfound: 
1293                      # append Pointset of varname values at the t vals
 
1294                      val = trajseq[trajseq_pos](trel_part, coords,
 
1295                                                 asGlobalTime=False,
 
1296                                                 checklevel=1) 
1297                      if len(t) == 1: 
1298                          retvals = val.toarray().ravel() 
1299  ##                        try:
 
1300  ##                            retvals = val.ravel()
 
1301  ##                        except AttributeError:
 
1302  ##                            print "Trajectory.py WARNING line 1129 - wrong type found"
 
1303  ##                            retvals = val.toarray().ravel()
 
1304                      else: 
1305                          retvals.append(val) 
1306                  else: 
1307                      tinterval = self.indepdomain 
1308                      print "valid t interval:", \
 
1309                            tinterval.get() 
1310                      print "t in interval? -->", tinterval.contains(tval) 
1311                      print "interval abs eps = ", tinterval._abseps 
1312                      raise ValueError('t = '+str(tval)+' is either not in any '
 
1313                                         'time interval defined for this '
 
1314                                         'trajectory, or there was an out-of-range'
 
1315                                         ' value computed (see above warnings). '
 
1316                                         'Decrease step-size / event tolerance or'
 
1317                                         ' increasing abseps tolerance.') 
1318                  # No need to increment tix here. It was incremented inside
 
1319                  # the inner while loop the last time a tval was checked.
 
1320                  # A failed containment there left tix at the next new value
 
1321              # Only return coords contained in all trajectory segments, but after
 
1322              # switching back to user-domain hierarchical names (if used) the
 
1323              # coords list needs to be re-sorted to correspond to the already
 
1324              # sorted values in retvals
 
1325              coordnames = self._FScompatibleNamesInv(intersect( \
 
1326                                        trajseq[trajseq_pos].coordnames,
 
1327                                        coords)) 
1328              coordnames.sort() 
1329              if len(t) == 1: 
1330                  if len(coords) == 1: 
1331                      return retvals[0] 
1332                  else: 
1333                      return Point({'coordarray': retvals,
 
1334                            'coordnames': coordnames,
 
1335                             'norm': self._normord}) 
1336              else: 
1337                  return Pointset({'coordarray': concatenate(retvals).T,
 
1338                           'coordnames': coordnames,
 
1339                           'indepvartype': self.indepvartype,
 
1340                           'indepvararray': t,
 
1341                           'indepvarname': self.indepvarname,
 
1342                           'norm': self._normord})

1343  
 
1344  
 


1345 -    def sample(self, coords=None, dt=None, tlo=None, thi=None,
 
1346                 doEvents=True, precise=False, asGlobalTime=True):


1347          """Uniformly sample the trajectory over range indicated,
 
1348            including any event points specified by optional 'doEvents'
 
1349            argument (default True).
 
1350          Returns a pointset.
 
1351  
 
1352          precise=False attempts to use the underlying mesh of the trajectory
 
1353            to return a Pointset more quickly. Currently, this can only be used
 
1354            for trajectories that have a single segment.
 
1355  
 
1356          If dt is not given, the underlying time mesh is used, if available.
 
1357  
 
1358          asGlobalTime is ignored for this class (time is always global for
 
1359            this class).
 
1360          """ 
1361  
 
1362          if coords is None: 
1363              coords = self.coordnames 
1364          else: 
1365              if not isinstance(coords, list): 
1366                  assert isinstance(coords, str), \
 
1367                         "coords must be a list of strings or a singleton string" 
1368                  coords = [coords] 
1369              coords = self._FScompatibleNames(coords) 
1370          t_interval = self.indepdomain 
1371          if tlo is None: 
1372              tlo = t_interval[0] 
1373          if thi is None: 
1374              thi = t_interval[1] 
1375          if t_interval.issingleton: 
1376              t = t_interval.get() 
1377              # [t] ensures return of a singleton Pointset
 
1378              pset = self.__call__([t], coords) 
1379              pset.name = self.name + "_sample" 
1380              if doEvents: 
1381                  for evname, ev_ts in self.eventTimes.items(): 
1382                      if t in ev_ts: 
1383                          pset.addlabel(0, 'Event:'+evname, {'t': t}) 
1384              return pset 
1385          else: 
1386              assert tlo < thi, 't start point must be less than t endpoint' 
1387              if doEvents: 
1388                  evs_gen = self.eventTimes 
1389              else: 
1390                  evs_gen = None 
1391              if dt is not None: 
1392                  assert dt < abs(thi-tlo), 'dt must be smaller than time interval' 
1393  ##        evs_mod_struct = self.eventStruct
 
1394          # evs_mod = ?
 
1395  ##        if evs_mod or evs_gen:
 
1396          if precise: 
1397              if dt is None: 
1398                  raise ValueError("Must specify an explicit dt when precise flag"
 
1399                                   " is set") 
1400              tmesh = concatenate([arange(tlo, thi, dt), [thi]]) 
1401              if evs_gen: 
1402  ##                # combine event data dictionaries
 
1403  ##                evs = copy.copy(evs_gen)
 
1404  ##                if evs_mod != {}:
 
1405  ##                    evs.update(dict(evs_mod))
 
1406                  # sort into ordered list of times
 
1407                  evtlist = orderEventData(evs_gen, nonames=True) 
1408                  if evtlist != []: 
1409                      tmesh = insertInOrder(tmesh, evtlist) 
1410              if len(tmesh) > 0: 
1411                  pset = self(tmesh,coords) 
1412              else: 
1413                  return None 
1414          else: 
1415              pset = None 
1416              for traj in self.trajSeq: 
1417                  # use fact that Trajectory.sample automatically truncates
 
1418                  # tlo and thi if they are beyond that segment's defined range.
 
1419                  if pset is None: 
1420                      try: 
1421                          pset = traj.sample(coords, dt, tlo, thi, doEvents,
 
1422                                             False) 
1423                      except ValueError, e: 
1424                          if e.message[:7] in ('tlo too', 'thi too'): 
1425                              # tlo or thi out of range
 
1426                              pass 
1427                          else: 
1428                              raise 
1429                  else: 
1430                      try: 
1431                          pset_new = traj.sample(coords, dt, tlo, thi,
 
1432                                                  doEvents, False) 
1433                      except ValueError, e: 
1434                          if e.message[:7] in ('tlo too', 'thi too'): 
1435                              # tlo or thi out of range
 
1436                              pass 
1437                          else: 
1438                              raise 
1439                      else: 
1440                          pset.append(pset_new, skipMatchingIndepvar=True) 
1441          pset.indepvarname = self._FScompatibleNamesInv(pset.indepvarname) 
1442          pset.mapNames(self._FScompatibleNamesInv) 
1443          return pset

1444  
 
1445  
 
1446  # ---------------------------------------------------------------------
 
1447  
 


1448 -def findApproxPeriod(traj, t0, t1_guess=None, T_guess=None, coordname=None,
 
1449                 ttol=1e-5, rtol=1e-2, guess_tol=0.1):


1450      """findPeriod does not ensure minimum period (it is up to the user to
 
1451      select a good initial guess, but guess_tol fraction of T_guess will be
 
1452      checked as a bracketing interval for bisection.""" 
1453      if t1_guess is None and T_guess is None: 
1454          raise ValueError("Must supply guess for either t1 or period, T") 
1455      if t1_guess is None: 
1456          if T_guess <= 0: 
1457              raise ValueError("Guess for period T must be positive") 
1458          t1_guess = t0+T_guess 
1459      if T_guess is None: 
1460          T_guess = t0+t1_guess 
1461      if t1_guess <= t0: 
1462          raise ValueError("t1 guess must be greater than t0") 
1463      if coordname is None: 
1464          coordname = traj._FScompatibleNamesInv(traj.coordnames[0]) 
1465      p0v = traj(t0, coordname)[0] 
1466      def f(t): 
1467          try: 
1468              return traj(t, coordname)[0]-p0v 
1469          #except (PyDSTool_BoundsError, ValueError):
 
1470          #    r = 1000.*(t-t0)
 
1471          except: 
1472              print "Error at t=%f: "%t, sys.exc_info()[0], \
 
1473                          sys.exc_info()[1] 
1474              raise

1475      try: 
1476          result = bisect(f, t1_guess-guess_tol*(t1_guess-t0),
 
1477                          t1_guess+guess_tol*(t1_guess-t0),
 
1478                          xtol=ttol) 
1479      except RuntimeError: 
1480          raise ValueError("Did not converge for this initial guess") 
1481  
 
1482      if traj.dimension == 1: 
1483          max_rval = abs(traj(result) - p0v) 
1484          rval = max_rval/abs(p0v)  # used for error info 
1485      else: 
1486          xt0 = traj(t0) 
1487          val = traj(result)-xt0 
1488          rval = [abs(val[vn]/xt0[vn]) for vn in traj._FScompatibleNamesInv(traj.coordnames)] 
1489          max_rval = max(rval) 
1490      if max_rval<rtol: 
1491          return abs(result-t0) 
1492      else: 
1493          print "Did not converge. The endpoint difference at t=%f was:\n"%result, \
 
1494            repr(val), \
 
1495            "\nwith infinity-norm %f > %f tolerance.\n"%(max_rval,rtol), \
 
1496            "Try a different starting point,", \
 
1497            "a different test variable, or reduce relative tolerance." 
1498          raise ValueError("Did not converge") 
1499
```

  


| Home | Trees | Indices | Help | PyDSTool |
| --- | --- | --- | --- | --- |

|  |  |
| --- | --- |
| Generated by Epydoc 3.0.1 on Fri May 4 15:24:25 2012 | http://epydoc.sourceforge.net |
